# The status of glucocorticoid-induced leucine zipper protein in the salivary glands in Sjögren’s syndrome: predictive and prognostic potentials

**DOI:** 10.1186/s13167-016-0052-8

**Published:** 2016-02-05

**Authors:** Xu Qin, Jun Yao Liu, Rafik Abdelsayed, Xingming Shi, Jack C. Yu, Mahmood S. Mozaffari, Babak Baban

**Affiliations:** 1 Department of Oral Biology, Dental College of Georgia, Augusta University, Augusta, GA 30912 USA; 2 Department of Stomatology, Tongji Hospital, Tongji Medical College, Huazhong University of Science and Technology, Wuhan, 430030 China; 3 Department of Oral Health and Diagnostic Sciences, Dental College of Georgia, Augusta University, Augusta, GA 30912 USA; 4 Department of Orthopedic Surgery, Augusta University, Augusta, GA 30912 USA; 5 Section of Plastic Surgery, Department of Surgery, Medical College of Georgia, Augusta University, Augusta, GA 30912 USA

**Keywords:** GILZ, Sjögren’s syndrome, Salivary glands, Inflammation, Predictive preventive and personalized medicine

## Abstract

**Background:**

We recently showed that an imbalance between the pro-inflammatory cytokine, interleukin (IL)-17, and the developmental endothelial locus-1 (Del-1) likely contributes to inflammation and salivary gland abnormalities in Sjögren’s syndrome (SS). The glucocorticoid-induced leucine zipper (GILZ) protein is a pivotal player in mediating the anti-inflammatory effects of glucocorticoids. However, its status and role in salivary gland inflammation and dysfunction in SS are not established. Thus, we tested the hypothesis that SS is associated with reduced GILZ expression, thereby contributing to Del-1/Il-17 imbalance and inflammation in salivary glands.

**Methods:**

We utilized the nonobese diabetic (NOD) mice, a model of SS-like disease as well as lower-lip biopsy samples of subjects without or with a diagnosis of SS in association with immunostaining studies. These studies were complemented with in vitro and flow-cytometry studies whereby interleukin (IL)-23-treated salivary gland cells were co-cultured with GILZ-expressing cells or control cells; IL-23 is known to increase generation of IL-17.

**Results:**

Salivary glands of NOD mice displayed marked leukocyte infiltration and reduced GILZ expression in association with increased IL-17 but decreased Del-1 expression. A similar pattern was observed for lower-lip biopsy samples of SS than non-SS subjects. Further, IL-23-treated salivary gland cells displayed marked increase in IL-17 but reduced Del-1 positive cells which were reversed with co-culturing with GILZ-expressing cells but not control cells. Collectively, the results are suggestive of dysregulation of GILZ playing a role in inflammation and associated Del-1/Il-17 imbalance in SS.

**Conclusions:**

Complementing our demonstration of Del-1/IL-17 imbalance in salivary glands in SS, the present study has established the relevance and significance of GILZ as a novel predictive and prognostic molecular fingerprint for SS. Thus, assessment of minor salivary gland GILZ expression, in conjunction with Del-1/IL-17 imbalance, could potentially offer a more sensitive approach to help with diagnosis of SS, at early stage of the disease, than that based on leukocyte infiltration. Future studies should establish whether treatment with GILZ ameliorates signs and symptoms of salivary malfunction of SS for which effective treatment options remain elusive.

## Background

Sjögren’s syndrome (SS) is a systemic autoimmune disease with a prevalence of 1–3 %, affecting more women than men (ratio of 9:1). It is characterized by chronic focal leukocyte infiltration and inflammation of exocrine glands, primarily involving salivary (and lacrimal) glands thereby resulting in persistent dryness of the mouth (and eyes) [1–4]. The salivary gland dysfunction is of major consequence for oral health including increased susceptibility to dental caries, gingivitis, and periodontitis [5–7]. SS can occur as a clinical entity alone or co-expressed with other systemic autoimmune rheumatic disorders. The serological hallmark of SS is the presence of circulating autoantibodies against soluble nuclear RNA containing antigens, Ro/SSA, and La/SSB [1–4].

The etiology and pathogenesis of SS remain elusive but hallmark histopathological findings of the disease include infiltration of cells of the innate and adaptive immunity (e.g., macrophages, T and B cells, dendritic cells) around vascular and glandular ducts of exocrine glands [8–13]. Nonetheless, it is increasingly acknowledged that the salivary hypofunction of SS is not adequately explained based on immune cell infiltration alone. This notion is supported by observations that dysregulation of salivary function and signal transduction pathways can occur prior to focal inflammation and reduction in saliva production. Consequently, the “epitheliocentric” model has emerged which views epithelial cells as playing a pivotal role in the pathogenesis of SS [14]. Thus, it is essential to unravel the contribution of the endogenous mechanisms which regulate local tissue inflammatory environment and could also contribute to the recruitment of immune and inflammatory cells with consequent further exacerbation of the disease process. Similarly important is the investigation of endogenous regulatory mechanisms which curtail immune and inflammatory responses.

The glucocorticoid-induced leucine zipper (GILZ) is a glucocorticoid-induced transcriptional regulatory protein which is advanced as the critical factor regulating the anti-inflammatory effects of glucocorticoids [15–19]. It is plausible that GILZ, either directly or indirectly, mediates glucocorticoid-induced leukocyte adhesion and migration [20]. A potential candidate for mediating the effects of glucocorticoids/GILZ on leukocyte movement is the developmental endothelial locus-1 (Del-1) which is a critical regulator of leukocyte adhesion and migration [21–24]. Importantly, a reciprocal functional link exists between Del-1 and the pro-inflammatory cytokine, interleukin (IL)-17 [23–25]. We conjectured that a reduction in GILZ may represent a critical event likely leading to Del-1/IL-17 imbalance. Thus, we tested the hypothesis that SS is associated with reduced GILZ thereby leading to Del-1/IL-17 imbalance associated with immune and inflammatory cell infiltration causing exacerbation of salivary gland inflammation. For this investigation, we utilized salivary glands of nonobese diabetic (NOD) mice and their healthy controls as well as lower-lip biopsy samples of human subjects without or with a diagnosis of SS. Importantly, the advent of cells which overexpress GILZ can facilitate investigation of its role in salivary glands. Thus, as an initial approach and utilizing in vitro protocols, we also explored the impact of GILZ delivery on salivary gland cells of mice which were treated with the pro-inflammatory cytokine, IL-23, on Del-1/IL-17 imbalance; Il-23 is an upstream of IL-17 and is known to upregulate its expression [26].

## Methods

Female nonobese diabetic (NOD; NOD/ShiLtJ) and control (NON/ShiLtJ) mice were purchased from the Jackson laboratory (Bar Harbor, Maine). The animals were housed, in the laboratory animal facilities at the Georgia Regents University, with free access to food and water; the use of animals for these studies was approved by the Institutional Animal Care and Use Committee. At 14 weeks of age, the animals were sacrificed and the submandibular/sublingual salivary glands were procured and placed in buffered-formalin for subsequent hematoxylin-eosin (H&E) staining and immunohistochemistry.

In order to establish the relevance of findings from the animal model of SS for the human condition, we obtained archived cases of lower-lip biopsies which were documented to be consistent with SS and those without a diagnosis of SS; IRB approval was secured for the use of archived biopsy samples in our studies.

Tissue sections, 4 μm in thickness, were cut from formalin-fixed, paraffin-embedded salivary glands of NOD and control mice as well as from SS and non-SS subjects. These sections (of mice and/or human subjects) were subjected to H&E staining as well as immunohistochemical staining using antibodies for GILZ, Del-1, and Il-17; 4′,6-diamidino-2-phenylindole (DAPI) was used as a nuclear marker [27–29].

As an initial step to explore our postulate and in a “proof-of-concept” experiment, salivary gland cells (SGCs) were prepared from wild-type mice (*n* = 4) and treated with IL-23 (2 μg/ml) which is known to markedly increase IL-17 production [26]. Further, IL-23-treated SGCs were co-cultured with GILZ-expressing cells or control cells expressing the green fluorescent protein (5:1 ratio for SGCs and GILZ or control cells) for 18 h.; we conjectured that upregulation of IL-17 generation should result in decreased Del-1 (i.e., Del-1/IL-17 imbalance), effects which would be reversed by GILZ expressing, but not, control cells. Thereafter, the cells were subjected to flow cytometry for determination of percent of Del-1- and IL-17-positive cells; the average of triplicate samples for each of the four mice was recorded for each parameter. It is noteworthy that GILZ-expressing mesenchymal stem cells or those expressing the green fluorescent protein have been developed and characterized using Western blotting and immunofluorescent studies. Briefly, murine bone marrow-derived mesenchymal stem cells were initially isolated using a negative immune-depletion followed by a positive immune-selection procedure and characterized for their multi-lineage differentiation capacity [30, 31]. Thereafter, retroviral transfection protocols were carried out to generate GILZ-expressing cells or those expressing the green fluorescent protein; these cells have been utilized in a variety of studies (e.g., [30–32]). To complement our studies, we also carried out immunofluorescent studies to demonstrate the marked expression of GILZ in GILZ expressing than control cells expressing the green fluorescent protein.

### Statistics

Data are expressed as means ± SEM. All data were analyzed using the analysis of variance followed by the Newman Keuls post hoc test to establish significance (*p* < 0.05) among groups.

## Results

**Fig. 1:**
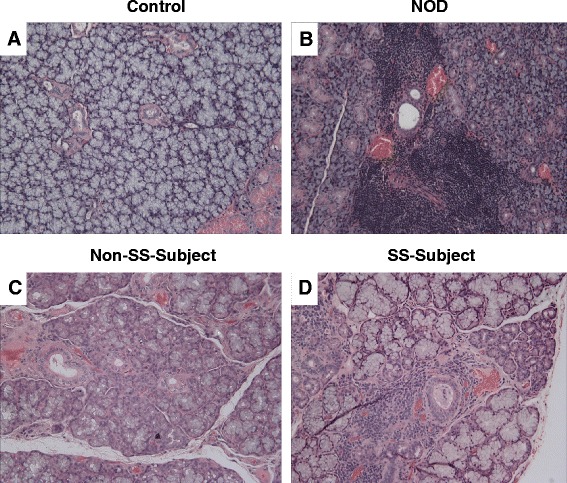
*Upper panels* (**a**, **b**) show representative H&E staining of salivary tissues from control and NOD mice while *lower panels* show representative H&E images for lower-lip biopsies of non-SS subject and SS subject (**c**, **d**). As expected, salivary tissue of NOD mouse and lower-lip biopsy of SS subject shows marked leukocyte infiltrations. ×200

Figure 1 shows the hallmark feature of leukocyte infiltration into salivary glands of NOD mice compared to their controls (panels a–b) as well as in lower-lip biopsy samples of subjects with a diagnosis of SS compared to those without a diagnosis of SS (panels c–d).

**Fig. 2:**
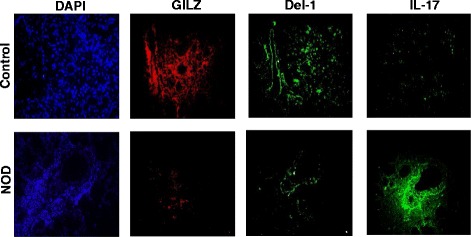
*Panels* show representative immunofluorescent images for GILZ, Del-1, and IL-17 for control and NOD mice; DAPI was used as a nuclear marker. ×1000

Next, we examined the status of GILZ in relation to Del-1and IL-17 in the salivary glands of control and NOD mice. Figure 2 shows representative images for 14-week-old control and NOD mice. As expected, paraffin-embedded salivary tissues of NOD mice displayed marked reductions in both GILZ and Del-1but a marked increase in IL-17 immunostainings.

**Fig. 3:**
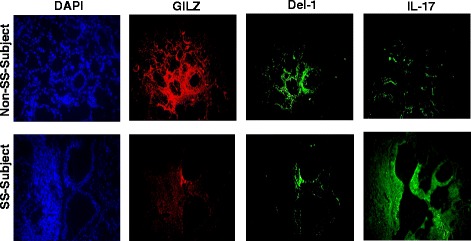
*Panels* show representative immunofluorescent images for GILZ, Del-1, and IL-17 of the salivary glands of non-SS and SS subjects; DAPI was used as a nuclear marker. ×1000

In order to explore the relevance of our observations using the NOD mouse model, we carried out immunostaining studies for GILZ, Del-1, and IL-17 on lower-lip biopsy samples of female patients with a diagnosis of Sjögren’s disease; lower-lip biopsy sample of patients for whom a diagnosis of SS was not made served as controls. As shown in representative images (Fig. 3), the biopsy sample of control patient showed prominent GILZ and Del-1 immunostainings, but samples of SS patients were minimally stained for either GILZ or Del-1. On the other hand, IL-17 immunostaining was very intense for lower-lip biopsy sample of SS patient than non-SS subject (Fig. 3).

**Fig. 4:**
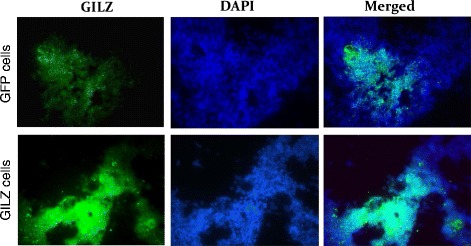
*Panels* show representative immunofluorescent images for GILZ in control cells (expressing the green fluorescent protein) or in GILZ-expressing cells; DAPI was used as a nuclear marker. ×1000

**Fig. 5:**
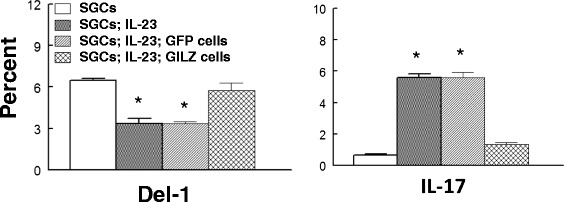
*Panels* show percent of cells which were positive for either Del-1 or IL-17 from in vitro studies whereby salivary gland cells (SGCs) were co-cultured with either control cells (expressing the green fluorescent) protein or GILZ-expressing cells; the culture medium lacked or contained IL-23 to stimulate IL-17 generation. *p < 0.05 compared to either SGCs or to SGCs; IL-23; GILZ-expressing cells

Representative immunofluorescent images of Fig. 4 show marked expression of GILZ in GILZ-expressing cells than control cells expressing the green fluorescent protein; these cells were used in subsequent in vitro studies. As shown in Fig. 5, treatment with IL-23 caused significant reductions in Del-1 but a marked increase in IL-17 positive cells. While control cells expressing the green fluorescent protein were largely without effects on these parameters, GILZ-expressing cells caused marked reversal of each parameter towards that of control SGCs, largely abrogating the impact of treatment with IL-23 and the associated increase in IL-17.

## Discussion

The present study shows that (a) GILZ is expressed in the major salivary glands of mouse as well as in minor salivary glands of the lower lip of human subjects, (b) the salivary glands of NOD mice as well as the lower-lip biopsy samples of SS subjects display marked reduction in GILZ expression, (c) the reduction in GILZ in salivary glands of both the NOD mice and SS subjects is associated with marked reduction in Del-1 but marked increase in IL-17, (d) the treatment of salivary gland cells with the pro-inflammatory cytokine, IL-23, markedly reduces Del-1 but increases IL-17 compared to their untreated controls, and (e) co-culturing of IL-23-treated salivary gland cells of mice with GILZ expressing, but not control, cells markedly increases Del-1 but reduces IL-17. Collectively, the results indicate a pivotal role for GILZ in the pathogenesis of salivary gland abnormalities of SS as well as a mechanistic link between GILZ and Del-1/IL-17 imbalance in salivary glands.

GILZ, also known as the tuberous sclerosis complex 22 (TSC22), is a recently described glucocorticoids (GCs)-induced transcriptional regulatory protein. The marked impact of GCs on GILZ is exemplified by studies showing that (a) dexamethasone markedly increases GILZ mRNA in human rheumatoid arthritis synovial fibroblasts [33], (b) in vivo, GCs increase GILZ expression while blockade of endogenous GCs inhibits its expression in the mouse spleen [16], and (c) GILZ expression is reduced in humans in response to reduced circulating cortisol [34]. The remarkable ability of GCs to induce GILZ expression relates to the direct binding of GC/glucocorticoid receptor complex to the six glucocorticoid-responsive elements located in the promoter region of the *GILZgene* [16, 17].

Another endogenous inhibitor of immune and inflammatory responses is Del-1 (also known as Edil3). Del-1 is a 53-kDa glycoprotein secreted by endothelial cells and associates with the endothelial cell surface and extracellular matrix. Del-1 expression was initially observed in embryonic cells including endothelial cells and thymus and subsequently shown in adult endothelial cells and some subsets of macrophages [21]. Further, Del-1 is expressed in adult mice in a tissue-specific manner with strong expression in the brain, eye, and lung [21]. Del-1 is shown to function as an endogenous inhibitor of a major leukocyte adhesion receptor, to prevent leukocyte adhesion to the endothelium thereby suppressing their entry to inflamed tissues [21–23]. Importantly, a reciprocal relation is established between Del-1 and IL-17 expression in the murine model of periodontitis and in the salivary glands in the setting of SS [23–25]. It is noteworthy that a recent study shows that the impact of IL-17 on downregulation of Del-1 involves a critical transcription factor, namely C/EBPβ (C/enhancer-binding protein) [35]. Accordingly, authors showed that IL-17 causes glycogen synthase kinase-3β (GSK-3β)-dependent phosphorylation of C/EBPβ, which is associated with diminished C/EBPβ binding to the Del-1 promoter and suppression of Del-1 expression. The inhibitory action of IL-17 was reversed at the GSK-3β level by phosphatidylinositol-4,5-bisphosphate 3-kinase/protein kinase B (Akt) signaling induced by D-resolvins. Importantly, the biological relevance of this regulatory network was confirmed in a mouse model of inflammatory periodontitis [35].

We now show the novel observation that GILZ expression is markedly reduced in the major salivary glands of NOD mice as well as in the minor salivary glands of human subjects with a confirmed diagnosis of SS compared to their respective controls. The reduction in GILZ was associated with the hallmark feature of SS (i.e., leukocyte infiltration of salivary tissues) as well as marked reduction in Del-1 but marked increase in IL-17 expression (i.e., Del-1/IL-17 imbalance). Collectively, these observations implicate a pathogenic role for GILZ in salivary gland abnormalities of SS and associated Del-1/IL-17 imbalance.

As an initial approach exploring potential mechanistic link between GILZ and Del-1/IL-17 imbalance, we carried out in vitro studies whereby salivary gland cells (SGCs) of wild-type mice were treated with IL-23 in order to increase IL-17 expression. We conjectured that the induced-IL-17 expression would be associated with reduced Del-1 expression which, in turn, would be abrogated with GILZ delivery using GILZ-expressing cells. Indeed, the results of flow cytometry studies confirmed that IL-23 treatment markedly increases IL-17 but reduces Del-1 expression. Further, while co-culturing of IL-23 treated SGCs with control cells expressing the green fluorescent protein was largely without effect on parameters of interest, co-culturing of SGCs with GILZ-expressing cells markedly reduced IL-17 but increased Del-1 thereby abrogating the Del-1/IL-17 imbalance.

## Conclusions

These observations suggest that GILZ likely regulates Del-1 expression in the salivary glands and prevents the deleterious impact of pro-inflammatory cytokine, IL-17, on Del-1 level. Our observations with the salivary glands are consistent with our recent demonstration that GILZ’s effect on the development of regulatory T cells in bone marrow mesenchymal lineage cells or bone marrow-derived mesenchymal stem cells involves the upregulation of Del-1. Further expression of Del-1 increases in the bone tissues of GILZ transgenic mice, and this increase is coupled with a significant increase in the production of anti-inflammatory cytokine, IL-10, but decrease in the production of pro-inflammatory cytokines, IL-6 and IL-12 [32]. In light of these observations, future studies should explore the effectiveness of GILZ delivery (e.g., via the use of GILZ-expressing cells on the course of SS in the animal model of the disease (i.e., NOD mouse) to establish its translational potential for the human condition.

### Perspective

The present study focused on the role of GILZ, in relation to Del-1/IL-17 imbalance, in salivary gland abnormalities of SS. Importantly, however, we suggest that unraveling of these mechanisms is of broader relevance and significance for SS. This contention is based on the fact that SS adversely impacts multiple organ systems including the lungs, kidneys, heart, gastrointestinal tract, genitourinary tract, and vascular and musculoskeletal systems. Most significantly, there is the dreaded complication of the development of malignancies and in particular, lymphoma [8–14]. Thus, the ability to modulate GILZ could provide the opportunity to not only address oral health consequences of salivary hypofunction (i.e., dental caries, gingivitis, and periodontitis) but could lead to gene-based therapies to address other grave systemic consequences of SS. Further, the molecular fingerprints which we have focused on in this study could individually and/or in combination be exploited as novel diagnostic and prognostic indicator(s) for SS. Thus, the findings of the present study are of relevance and significance for predictive, preventive, and personalized medicine (PPPM) which has emerged as an alternative approach the more conventional, reactionary, practice of medicine [36]. The central tenet of PPPM proposes that early detection and prevention of disease while tailoring care to the needs of the individual patient are more conducive to a healthier population accompanied with reduced cost of care than the often costly treatment of complications of a fully manifest disease. Importantly, however, effective implementation of PPPM is contingent upon the unraveling of pathogenic mechanisms of the disease, an aspect which we have focused on this report as it relates to salivary gland abnormalities of SS.

## Abbreviations

Del-1: developmental endothelial locus-1
GCs: glucocorticoids
GILZ: glucocorticoid-induced leucine zipper
IL: interleukin
NOD: nonobese diabetic
SGCs: salivary gland cells
SS: Sjögren’s syndrome
TSC22: tuberous sclerosis complex 22
